# Photo-Crosslinked Pro-Angiogenic Hydrogel Dressing for Wound Healing

**DOI:** 10.3390/ijms25189948

**Published:** 2024-09-15

**Authors:** Wang Zhang, Shuyi Qian, Jia Chen, Tianshen Jian, Xuechun Wang, Xianmin Zhu, Yixiao Dong, Guoping Fan

**Affiliations:** 1 Shanghai Institute for Advanced Immunochemical Studies (SIAIS), ShanghaiTech University, Shanghai 201210, China; zhangwang@shanghaitech.edu.cn (W.Z.); qianshy@shanghaitech.edu.cn (S.Q.); chenjia1@shanghaitech.edu.cn (J.C.); jiantsh@shanghaitech.edu.cn (T.J.); wangxch2@shanghaitech.edu.cn (X.W.); zhuxm@shanghaitech.edu.cn (X.Z.); 2 Shanghai Clinical Research and Trial Center, ShanghaiTech University, Shanghai 201210, China; 3 School of Life Science and Technology, ShanghaiTech University, Shanghai 201210, China; 4 Shanghai Academy of Sciences & Technology Institute of Model Animals Transformation, Shanghai 201203, China

**Keywords:** burn, hydrogel, methacrylate hyaluronic acid, prominin-1-binding peptide, vascular endothelial growth factor, wound healing

## Abstract

Severe burns are one of the most devastating injuries, in which sustained inflammation and ischemia often delay the healing process. Pro-angiogenic growth factors such as vascular endothelial growth factor (VEGF) have been widely studied for promoting wound healing. However, the short half-life and instability of VEGF limit its clinical applications. In this study, we develop a photo-crosslinked hydrogel wound dressing from methacrylate hyaluronic acid (MeHA) bonded with a pro-angiogenic prominin-1-binding peptide (PR1P). The materials were extruded in wound bed and in situ formed a wound dressing via exposure to short-time ultraviolet radiation. The study shows that the PR1P-bonded hydrogel significantly improves VEGF recruitment, tubular formation, and cell migration in vitro. Swelling, Scanning Electron Microscope, and mechanical tests indicate the peptide does not affect the overall mechanical and physical properties of the hydrogels. For in vivo studies, the PR1P-bonded hydrogel dressing enhances neovascularization and accelerates wound closure in both deep second-degree burn and full-thickness excisional wound models. The Western blot assay shows such benefits can be related to the activation of the VEGF–Akt signaling pathway. These results suggest this photo-crosslinked hydrogel dressing efficiently promotes VEGF recruitment and angiogenesis in skin regeneration, indicating its potential for clinical applications in wound healing.

## 1. Introduction

The skin is the largest organ of the human body and serves as the first line of defense against the external environment [1]. Naturally, skin has a certain level of self-healing capability for small injuries and lesions. However, large-area trauma and pathological substances can disrupt the healing process, leading to chronic wounds and even threatening lives [2]. As an example, severe burns are one of the most traumatic injuries that can cause significant morbidity and mortality [3,4,5]. Many wound dressings and skin grafts have been developed to benefit severe burn patients by protecting against infection and reducing mortality [6]. Yet, ischemia and sustained inflammation often result in slow wound healing, infection, and hypertrophic scarring, which remain the major challenges in burn management [7]. 

Vascular endothelial growth factor (VEGF) has been widely acknowledged as a crucial angiogenic growth factor that plays a pivotal role in promoting angiogenesis and epithelialization in wound healing [8,9]. Nevertheless, due to the short half-life and instability, the potential for clinical applications of VEGF remains unsatisfied [10,11,12]. To solve this problem, many delivery systems are designed for the controlled release of VEGF, such as nano/micro-particles, liposomes, and hydrogels [13,14,15]. In addition, a variety of recombinant peptides have been constructed targeting VEGF and its receptors to modulate their angiogenic responses. As one of the pioneers, D’Andrea et al. developed a short peptide that reproduced the VEGF 17–25 helix region, which can specifically bind to the VEGF receptor and promote capillary formation [16]. More recently, Adini et al. reported the prominin-1-binding peptide (PR1P) derived from the pro-angiogenic glycoprotein [17]. Previous studies have demonstrated that the PR1P promotes neovascularization for myocardial infarction, patellar ligament regeneration, and cutaneous wound healing [18,19,20]. Most of these studies injected PR1P in wound area directly or mixed within a scaffold, leading to reduced maintainability and functionality. 

**Figure 1:**
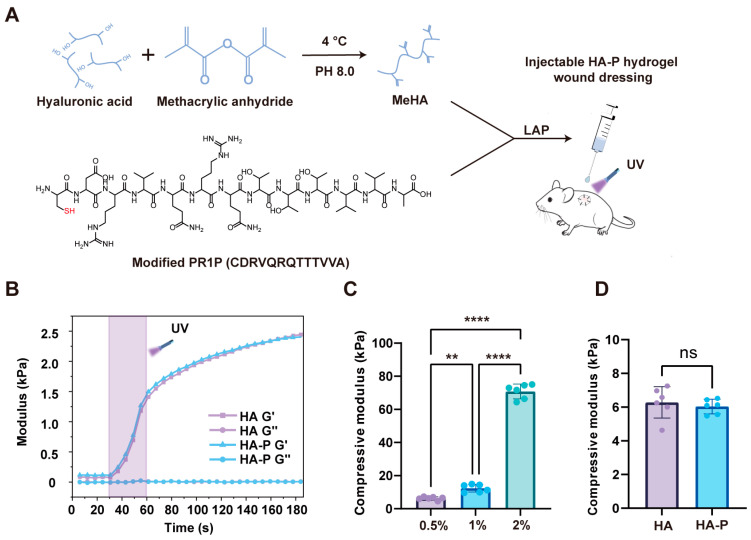
Fabrication of an injectable HA-P hydrogel wound dressing. (**A**) Schematic illustration of the synthesis of MeHA and in situ crosslinking with cysteine-modified PR1P to form a hydrogel wound dressing. (**B**) Real-time crosslinking rheological measurements of HA and HA-P hydrogels (0.5% *w*/*v*) with 30 s exposure to UV radiation. (**C**) Compressive modulus of HA hydrogels with different material concentrations (gelation with 30 s exposure to UV radiation). (**D**) Compressive modulus of HA and HA-P hydrogels (0.5% *w*/*v*, with 30 s exposure to UV radiation) (mean ± SD, n = 6, ** p < 0.01, **** p < 0.0001, ns, not statistically significant).

To further enhance the VEGF-recruiting efficiency, herein, we conjugated a cysteine-terminal PR1P with a methacrylate hyaluronic acid (MeHA) polymer and in situ formed a hydrogel wound dressing via a photo-crosslinking approach (Figure 1A). The injectable hydrogel can easily cover the irregular wound area. The PR1P covalently linked to the hydrogel backbone which helps to maintain the recruited VEGF. Along with the hydrogel degradation, VEGF can be sustainably released and benefit wound healing. In this study, the mechanical and physical properties of the HA-based hydrogel (HA) were fully characterized, and PR1P-bonded hydrogel (HA-P) showed a pro-angiogenic response in vitro. In addition, the HA-P hydrogel dressing significantly accelerated wound healing with increased angiogenesis and reduced scar formation, in both a deep second-degree burn model and a full-thickness excisional wound model. 

## 2. Results

### 2.1. Fabrication and Characterization of HA and PR1P-Bonded Hydrogels

To achieve the photo-crosslinkable MeHA polymer, we first functionalized HA (90–100 kDa) with methacrylic anhydride as previously reported [21]. The successful conjugation of methacrylic groups was confirmed by ^1^H NMR and FTIR analysis (Figures S1 and S2). The gelling precursor of MeHA was prepared in Dulbecco’s phosphate-buffered saline (DPBS) containing 0.25% (*w*/*v*) of lithium phenyl-2,4,6-trimethylbenzoylphosphinate (LAP) as a photo-initiator and crosslinked under exposure of ultraviolet (UV) radiation. The rheological measurement showed a rapid increase in the storage modulus (G’) under the UV radiation indicating the gelation occurring (Figure 1B). Then, we evaluated the mechanical strength of the photo-crosslinked HA hydrogel with different polymer concentrations and exposure time to UV radiation. As expected, the hydrogel strength was significantly enhanced with increased polymer concentration and prolonged exposure time (Figure 1C and Figure S3). For example, the compressive modulus of HA (0.5% *w*/*v*) hydrogel was about 6.3 ± 0.9 kPa after 30 s exposure to UV radiation, which increased to 10.8 ± 0.9 kPa with 90 s exposure and 70.8 ± 4.4 kPa for 2.0% (*w*/*v*) of HA hydrogel with 30 s exposure. Meanwhile, due to the abounding methacrylic groups on the MeHA polymer, the introduction of PR1P did not visibly change the mechanical properties of the hydrogels (Figure 1D).

**Figure 2:**
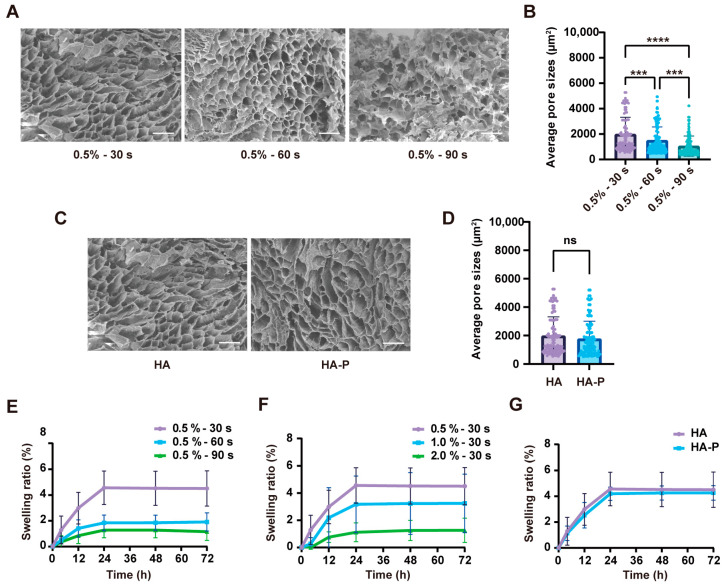
Characterization of HA and HA-P hydrogels. (**A**,**B**) SEM micrographs and quantification of the average pore size of freeze-dried HA hydrogels (0.5% *w*/*v*) with 30 s, 60 s, and 90 s of UV exposure. (**C**,**D**) SEM micrographs and quantification of the average pore size of HA and HA-P hydrogels (0.5% *w*/*v*, with 30 s of UV exposure). (**E**,**F**) Swelling ratios of HA hydrogels with various UV exposure times and material concentrations. (**G**) Swelling ratios of HA and HA-P hydrogels (0.5% *w*/*v*, with 30 s of UV exposure) (mean ± SD, n = 3, *** *p* < 0.001, **** p < 0.0001, ns, not statistically significant, scale bar, 100 μm).

To efficiently recruit and release VEGF to the wound site, it was important to assess the interconnected structure and swelling property of the hydrogels. The scanning electron microscopy (SEM) images showed the obvious porous morphology of the freeze-dried hydrogels (Figure 2A), and an increased exposure time decreased the average pore size (Figure 2B). Moreover, there was no significant difference in the pore size between HA and PR1P-bonded hydrogels (HA-P) (Figure 2C,D). For the swelling assay, the hydrogels reached a plateau after 24 h. It decreased in the hydrogels with a prolonged exposure time and an increased material concentration, due to the higher crosslinking density (Figure 2E,F). Similar to the above assessments, the addition of PR1P did not change the swelling property of the HA hydrogel (Figure 2G). Together, these results demonstrated stable HA hydrogels can be achieved with a tunable crosslinking density and mechanical properties, and adding PR1P would not influence the hydrogel features. To mimic the mechanical properties of cutaneous tissue, we chose the gelling formula of 0.5% (*w*/*v*) MeHA with 30 s UV exposure for the following experiments [22].

### 2.2. PR1P-Bonded Hydrogel Promoted VEGF Recruitment and Angiogenesis Response In Vitro

**Figure 3:**
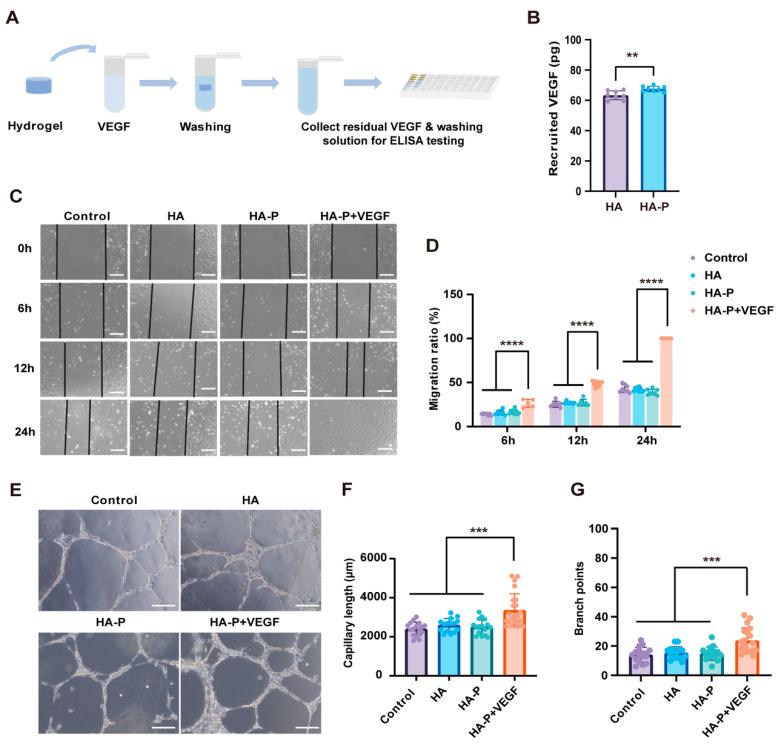
VEGF recruitment and in vitro angiogenic effect of HA-P hydrogels. (**A**) Schematic illustration of VEGF recruitment assay. (**B**) Quantitative analysis of the maintained VEGF within hydrogels shows the HA-P hydrogel binds more VEGF than HA hydrogel does (n = 8). (**C**) Representative images of cell migration in a scratch wound healing assay after 0, 6, 12, and 24 h. (**D**) Quantitative analysis of the migration ratio shows HA-P hydrogel loaded with VEGF significantly promotes cell migration compared with the other groups. (**E**) Representative images of the tube formation of HUVECs. (**F**,**G**) Quantitative analysis of capillary length and the number of branch points of the tubule network. The capillary length and branch points in HA-P hydrogels are significantly higher than in the other groups (mean ± SD, n = 3, ** p < 0.01, *** *p* < 0.001, **** *p* < 0.0001, scale bar, 200 μm).

Next, we detected the VEGF recruiting efficiency of the hydrogels by using a VEGF ELISA kit (Figure 3A). As a result, it was found more VEGF was maintained in the HA-P hydrogel than in the HA hydrogel (Figure 3B). To test whether the VEGF-loaded hydrogel provides angiogenic functionality, we performed the scratch wound healing assay using human umbilical venous endothelial cells (HUVECs). HA-P hydrogel loaded with VEGF was placed in a Transwell^®^ insert soaking in the culture medium. It was found that VEGF can be released from the hydrogel and it significantly promoted the recovery of the scratched wounds compared to the control and the hydrogels without loading VEGF (Figure 3C,D). In addition, the tube formation assay showed the increased capillary length and branch points caused by the released VEGF from the HA-P hydrogel (Figure 3E–G). Overall, these data indicated that the HA hydrogel linked with PR1P can efficiently bind and release VEGF, which can promote cell migration and angiogenesis in vitro.

### 2.3. PR1P-Bonded Hydrogel Promoted Wound Healing in Deep Second-Degree Burns

**Figure 4:**
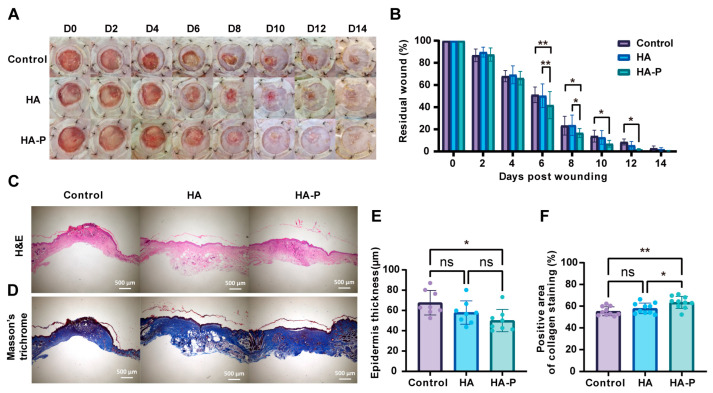
HA-P hydrogel dressing promotes wound regeneration in burns. (**A**) Representative photos exhibit the wound healing process. (**B**) Quantitative analysis of residual wound area (%) up to 14 days. HA-P hydrogel treatment shows significant acceleration of healing compared to the control group after day 6. (**C**) Representative images of H&E staining and (**D**) Masson’s trichrome staining of the wounds at 14 days post-wounding (scale bar, 500 μm). (**E**) Quantitative analysis of epithelium thickness and (**F**) collagen density indicates less epidermis hyperplasia and increased collagen deposition in the HA-P hydrogel treatment group (mean ± SD, n = 8–10, * p < 0.05, ** p < 0.01, ns, not statistically significant).

**Figure 5:**
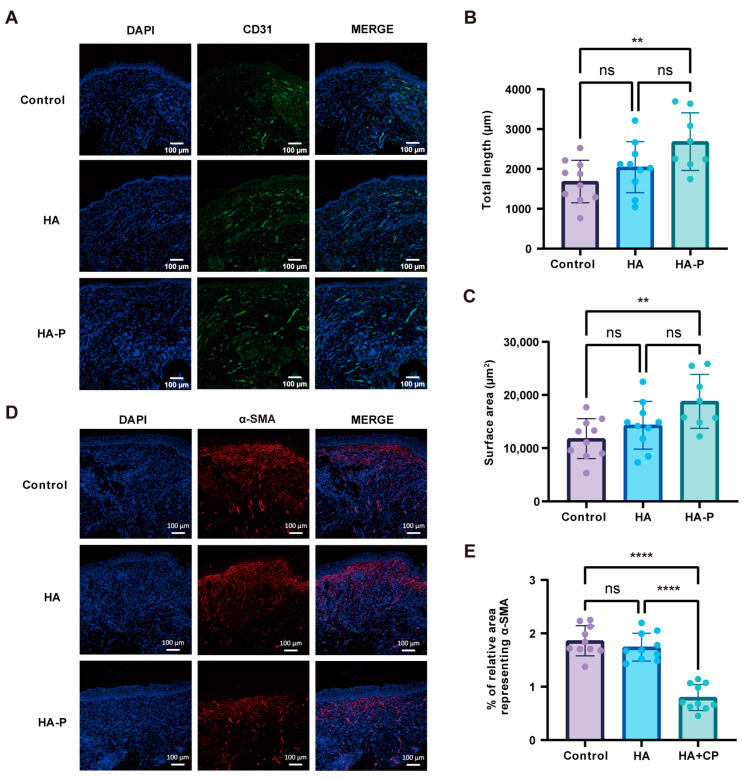
HA-P hydrogel dressing enhances angiogenesis and reduces myofibroblasts in burns. (**A**) Representative images of the CD31^+^ staining (green) of different groups at day 14 post-wounding. The nucleus was stained with DAPI (blue). (**B**,**C**) Stereological quantification of the surface area and length density of vasculature demonstrates a significant enhancement in angiogenesis for HA-P hydrogel compared with the control group. (**D**) Representative images of α-SMA^+^ staining (red) at day 14 post-wounding. The nucleus was stained with DAPI (blue). (**E**) Quantitative analysis of the positive area of α-SMA shows the HA-P hydrogel treatment significantly reduces myofibroblasts’ regeneration (mean ± SD, n = 8–10, ** p < 0.01, **** *p* < 0.0001, ns, not statistically significant, scale bar, 100 μm).

**Figure 6:**
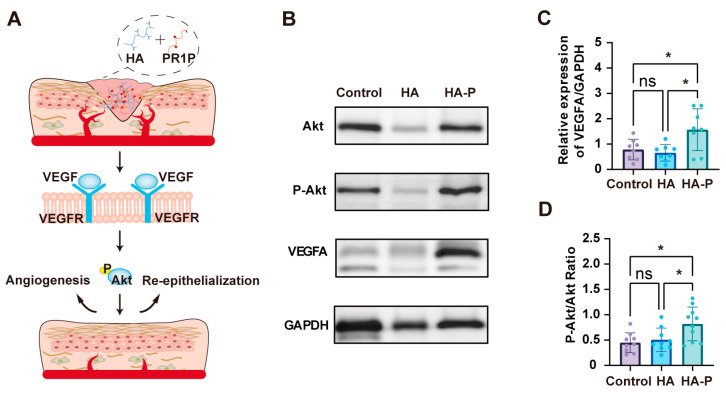
HA-P hydrogel dressing promoted angiogenesis via activation of the VEGF–Akt signaling pathway. (**A**) Schematic illustration of the molecular mechanism for HA-P hydrogel dressing which activates the VEGF–Akt signaling pathway in wound healing. (**B**) Representative images of Western blotting of Akt, p-Akt, and VEGFA in wounds at day 14 post-wounding. (**C**,**D**) Quantitative results of Western blotting show that the HA-P hydrogel treatment significantly increases the relative protein expression level of VEGFA and the relative expression ratio of p-Akt/Akt (mean ± SD, n = 8–10, * p < 0.05, ns, not statistically significant).

To evaluate the therapeutic efficacy of a PR1P-bonded hydrogel dressing in vivo, we performed wound healing assessments in a deep second-degree burn mouse model [23]. Compared with the control groups of “no treatment” and HA hydrogel, treating with HA-P hydrogel showed a significant acceleration of wound closure from day 6 post-wounding (Figure 4A,B). These significances were not so obvious between groups after 10 days, probably due to the wound contraction effect. At 14 days post-wounding, we collected the tissue samples for the histological analysis, when most wounds had healed completely in all groups. Hematoxylin and eosin (H&E) staining demonstrated re-epithelialization at 14 days in all groups (Figure 4C). Also, no obvious hydrogel fragments were found in the wound area, which indicated the HA-based hydrogels were fully degraded at this point. The quantitative analysis showed a thinner epidermis for the HA-P hydrogel group than the control group, suggesting less epidermis hyperplasia (Figure 4E). In addition, the Masson’s trichrome (MT) staining showed increased collagen deposition for the HA-P hydrogel group, indicating the promoted regeneration of granulation tissue (Figure 4D,F). Then, neoangiogenesis and myofibroblasts were determined by the immunofluorescence staining of CD31 and α-SMA. Visually scanning revealed more vasculature in the wound area for the HA-P hydrogel group than the control groups (Figure 5A). The quantification of the stereological analysis showed that both the surface area and length density of vasculature for the HA-P hydrogel group significantly increased compared to the no-treatment control (Figure 5B,C). Meanwhile, wounds treated with HA-P hydrogel exhibited a significant reduction in the α-SMA level, indicating that the treatment might inhibit myofibroblasts’ regeneration and scar formation (Figure 5D,E). To investigate the mechanism of our treatment promoting angiogenesis and wound healing, we performed a Western blot analysis using tissue samples focusing on the VEGF-related signaling pathway (Figure 6A). As a result, the expression level of VEGFA was enhanced in the HA-P hydrogel group (Figure 6B,C). In addition, with a similar expression level of total Akt, the HA-P hydrogel group exhibited an increased expression of phosphorylated Akt (p-Akt) (Figure 6D). Taken together, these results indicate that HA-P hydrogel dressing enhanced neovascularization and promoted wound healing in burns, probably via the activation of the VEGF–Akt signaling pathway. 

### 2.4. PR1P-Bonded Hydrogel Dressing Promoted Wound Regeneration in Acute Wounds

**Figure 7:**
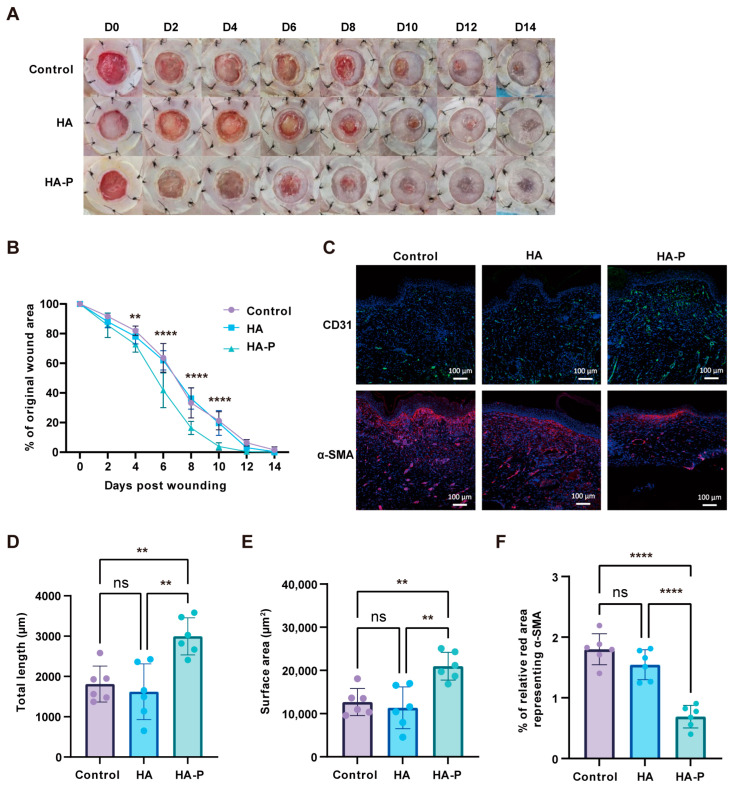
HA-P hydrogel dressing promotes wound healing in a full-thickness excisional wound model. (**A**) Representative images of the healing process up to 14 days post-wounding. (**B**) Wound closure curves of different groups show a significant acceleration of healing with the HA-P hydrogel treatment compared to the HA hydrogel and control group from day 4. (**C**) Representative images of CD31^+^ (green) and α-SMA^+^ (red) staining at day 14 post-wounding. The nucleus was stained with DAPI (blue). (**D**,**E**) Quantitative analysis indicates the HA-P hydrogel treatment significantly improves the angiogenesis and (**F**) reduces myofibroblasts’ regeneration (mean ± SD, n = 6, ** *p* < 0.01, **** *p* < 0.0001, ns, not statistically significant, scale bar, 100 μm).

Next, we wonder whether the PR1P-bonded hydrogel dressing can be applied to other types of wounds. Thus, we evaluated the therapeutic effects of the hydrogel dressing in a full-thickness excisional wound model. Similar to the burn model, we observed significant improvement in the healing process by HA-P hydrogel treatment, including a shorter healing time, less epidermis hyperplasia, and increased collagen deposition (Figure 7A,B and Figure S4). In addition, CD31 and α-SMA staining indicated that the HA-P hydrogel dressing significantly enhanced angiogenesis and reduced the infiltration of myofibroblasts compared to the other groups (Figure 7C–F). These results demonstrated the PR1P-bonded hydrogel can also benefit normal acute wounds.

## 3. Discussion

In this study, we utilize a photo-crosslinked pro-angiogenic hydrogel dressing for cutaneous wound healing. With a VEGF binding sequence, a cysteine-modified PR1P is conjugated within the HA-based hydrogel, which shows enhanced angiogenesis and accelerated healing in a deep second-degree burn model and a full-thickness excisional wound model. 

Hyaluronic acid (HA) is a major glycosaminoglycan content of extracellular matrix that is widely distributed in skin, joints, and cornea [24]. It exhibits excellent moisturizing properties and participates in numerous physiological processes, such as inflammatory responses and wound healing [25]. It has been demonstrated HA plays an important role in promoting cell proliferation and reducing scar formation [26,27]. However, the natural HA molecule has a weak mechanical strength and rapid degradation features that limit its application. To solve this problem, methacrylated hyaluronic acid (MeHA) has been widely used to fabricate a photo-polymerized hydrogel network with controlled mechanical behavior [21,28]. In this study, we conjugate methacrylic anhydride onto HA with a relatively small molecular weight (90–100 kDa), which has been reported for its pro-angiogenic and pro-regeneration functions [29]. With the presence of the LAP as a photo-initiator, the gelling precursor of the MeHA polymer can form a stable hydrogel dressing in situ under a short-time exposure to UV radiation. The injectable behavior makes this hydrogel dressing easy to use to cover the irregular wound shape and large wound area. To mimic the mechanical properties of normal skin tissue [22], we chose a hydrogel formula of 0.5% MeHA with a 30 s UV exposure time in our study, which exhibits a compressive modulus around 6.3 kPa (Figure 1C). In addition, we introduce a pro-angiogenic peptide in which the thiol group in cysteine can covalently bind to the vinyl group in MeHA during the gelation. Due to the excessive methacrylate group on the MeHA, conjugation of the peptide does not affect the physical and mechanical properties of the HA hydrogel (Figure 2). On the other hand, photo-crosslinked materials are often concerned with the safety issue of using UV light for clinical applications. Nevertheless, by controlling the exposure time and radiation density, UV-crosslinkable materials such as MeHA and GelMA (gelatin methacryloly) have been widely used in the tissue engineering field [30]. In addition, other photo-crosslinked hydrogels induced by visible light have been developed to further reduce the toxicity risks [31]. 

Severe burns are a common injury in clinics which can be life-threatening with deficient wound management and infections [32]. Sustained inflammation and ischemia in burns delay the healing process and significantly increase the infection risk. Previous reports have widely indicated that VEGF plays an important role in promoting the migration and proliferation of endothelial cells for neovascularization and re-epithelialization [33]. Nevertheless, the short half-life and concentration-dependent feature of VEGF treatment may result in the failure of its clinical application. PR1P is a short peptide sequence derived from the pro-angiogenic glycoprotein of prominin-1 [17]. It contains an extracellular VEGF-binding domain and has been demonstrated as a pro-angiogenic and immunomodulator in wound healing [12,34]. Most previous reports simply mixed PR1P within a scaffold, which leads to a burst decline of the peptide in the wound bed and reduces the VEGF recruiting efficiency [19]. In this study, we covalently conjugate PR1P to MeHA during gelation. Consequently, recruited VEGF assembles within the hydrogel and sustainably releases in the wound area along with the hydrogel degradation. In vitro assessments confirm that the PR1P-bonded hydrogel (HA-P) enhances the VEGF retention (Figure 3B). More importantly, using the Transwell^®^ system, our results show that the VEGF released from the HA-P hydrogel significantly promotes the migration (Figure 3C,D) and vascular tube formation of endothelial cells (Figure 3E–G). 

To estimate the therapeutic efficacy of an HA-P hydrogel dressing in wound healing, we first establish a deep second-degree burn model with heated metal rods [35]. The necrotic tissue is debrided by a biopsy punch two days post-burning. To prevent contraction in the rodent skin and evaluate the wound closure more accurately, we suture the circular silicone splints surrounding the wound area [36,37]. The hydrogel dressings are applied in situ with or without loading PR1P. Generally, for acute wound healing in human skin, vascularization occurs a few days post-injury and essentially contributes to the healing process. In severe burns, however, angiogenesis can be markedly reduced due to vasoconstriction and cause aggravated necrosis [38]. In this study, a significant acceleration of wound closure is observed after 6 days between the HA-P treatment and control groups (Figure 4B). Meanwhile, the results of immunofluorescence staining and stereological analysis confirm the enhanced angiogenesis in the treatment group (Figure 5). To reveal the mechanism of this therapeutic improvement, we perform the Western blot assay on the VEGF-related signals. Consistent with previous reports, the introduction of PR1P enhances angiogenesis by activating the VEGF–Akt signaling pathway [18]; in our study, the HA-P hydrogel treatment increases the expression level of VEGFA and phosphorylated Akt (Figure 6C,D). Furthermore, we expand the application of the HA-P injectable hydrogel dressing to normal acute wounds. Similar therapeutic outcomes are observed in a full-thickness excisional wound model. Interestingly, the improvement in wound closure, neovascularization, and scarring formation by the HA-P treatment seems more obvious in excisional wounds compared to burn wounds. We assume it is due to the ischemic condition in burn wounds restricting the therapeutic efficiency of the dressing [39]. Therefore, for future study, an HA-P hydrogel dressing combined with a VEGF delivery system might be worth investigating. 

## 4. Materials and Methods

### 4.1. Materials

Sodium hyaluronic acid (HA, 90–100 kDa) was purchased from Yuanye Bio-Technology Co., Ltd. (Shanghai, China). The cysteine-modified PR1P (CDRVQRQTTTVVA, purity ≥ 95%) was purchased from the GenScript Biotech Corporation (Nanjing, China). Other generally used chemicals were purchased from Sigma-Aldrich (St. Louis, MO, USA).

### 4.2. Synthesis of Methacrylate Hyaluronic Acid and Fabrication of the Hydrogels

Methacrylate hyaluronic acid (MeHA) was synthesized according to the procedure previously reported [21]. Briefly, 1 g of HA (90–100 kDa) was dissolved in 100 mL ultrapure water, and then 5 mL of methacrylic anhydride was added into the HA solution with stirring. The pH was adjusted to 8.0 by using 5 M NaOH and reacted on ice with stirring overnight. Then, the product was dialyzed (MW cutoff 8 kDa) against deionized water for 4 days, followed by lyophilization. The methacrylation was analyzed by ^1^H NMR (Bruker Avance III HD, Bruker BioSpin AG, Fällanden, Switzerland) and ATR-FTIR (Attenuated Total Refraction-Fourier transform infrared, Vertex 70, Bruker BioSpin AG). For the FTIR test, the scan ranged from 600 to 4500 cm^−1^ with a resolution of 8 cm^−1^ and a number of scans of 64. To fabricate the hydrogels, MeHA polymer, photo-initiator of Lithium phenyl-2,4,6 trimethylbenzoylphosphinate (LAP), and PR1P were dissolved in Dulbecco’s phosphate-buffered saline (DPBS, Thermo Fischer Scientific, Waltham, MA, USA) separately. The gelling precursor was prepared by mixing the solutions at the final concentration of MeHA from 0.5% to 2% (*w*/*v*) and LAP at 0.25% (*w*/*v*). Then, the gelling precursor was exposed to UV light (405 nm; 25 mW/cm^2^, Engineering for Life, Suzhou, China) for the desired time to afford HA hydrogels. For the HA-P hydrogel, the concentration of the PR1P was consistent at 0.2 mg/mL.

### 4.3. Characterization of the Hydrogels

The photo-crosslinking behavior of the hydrogel was tested by using a rheometer (DHR-2, TA Instruments, New Castle, DE, USA) with a steel parallel-plate geometry. An oscillatory-time sweep was performed at room temperature, with 1% strain and consistent frequency at 1 Hz. The UV light (405 nm) was initiated for 30 s for the gelation. The compressive modulus of hydrogels was determined by using a Universal Material Testing Machine (INSTRON 5966, Instron GmbH, Darmstadt, Germany). The hydrogels were swollen in DPBS for 24 h before the testing. The measurement was performed at a maximum strain of 80%, and the compressive modulus was calculated as the slope of the linear regions on the stress–strain curves. The swelling assessment of the hydrogels was performed at 37 °C. Hydrogels of 0.5% (*w*/*v*) of HA and HA-P were fabricated by 30 s of UV exposure. The initial weights of the hydrogel samples were measured as W_0_. After swelling in DPBS, the weight of each sample was recorded as W_t_ at different time points. The swelling ratio was determined using the following equation (Equation (1)):

Swelling ratio = (W_t_ − W_0_) /W_0_ × 100% (1)

Scanning electron microscopy (SEM) was used to observe the porous structure of the hydrogels. The hydrogels were frozen in liquid nitrogen followed by lyophilization. The freeze-dried samples were coated with a gold layer and imaged with Carl Zeiss Sigma 300 SEM (Carl Zeiss Microscopy GmbH, Oberkochen, Germany) at 10 kV. The porosity of the hydrogels was examined by using Image J software (v1.53k, National Institutes of Health, Washington, DC, USA).

### 4.4. In Vitro Pro-Angiogenic Assays

The loading efficiency of the VEGF in the hydrogel was determined using an ELISA kit [12]. Briefly, HA hydrogel (0.5% *w*/*v*) was prepared with 30 s of UV exposure as described above. The hydrogel samples were incubated in 0.4 ng/mL of VEGF solution for 8 h at 37 °C, followed by rinsing three times using DPBS with the washing solution collected. The residual VEGF was detected by the Mouse VEGF ELISA Kit (Solarbio^®^, Beijing, China) with the recommended procedure. 

The scratch wound healing assay was performed using human umbilical venous endothelial cells (HUVECs) with previously reported methodology [20]. The cells were cultured with EGM^TM^-2 medium (Lonza, Basel, Switzerland) in a humidified incubator at 37 °C with 5% CO_2_ until confluent monolayers were formed. Then, the cell monolayer in each well was scratched by using a pipette tip (200 μL) and the cell debris gently washed from the scratch with DPBS. To prepare the VEGF-loaded hydrogel sample, HA-P hydrogel was incubated in low-serum culture medium of DMEM/F12 (HyClone, Logan, UT, USA) with 2% fetal bovine serum (FBS, Bioind, Kibbutz Beit Haemek, Israel), supplemented with 25 ng/mL VEGF (rhVEGF165, NovoProtein, Suzhou, China) at 37 °C for 6 h, followed by washing out the surface-adherent protein with the medium. Then, HA, HA-P, and HA-P + VEGF hydrogels were loaded in a Transwell^®^ insert (Corning^®^, Corning, NY, USA) co-incubated with the scratched cells, compared to a control group without additional treatment. The gap area between the scratched cells was imaged from 0 to 24 h and measured by using Image J software. The cell migration ratio was calculated by using the following equation (Equation (2)), in which L_0_ represents the average distance of the initial scratched gap and L_t_ represents the average distance of the unhealed gap at each time point.

Migration ratio = (1 − L_t_/ L_0_) × 100% (2)

For the tube formation assay, HUVECs were seeded on a pre-coated culture plate with growth factor-reduced Matrigel^®^ (Corning^®,^ Corning, NY, USA) and cultured using EGM ^TM^-2 medium (Lonza, Basel, Switzerland) [40]. The hydrogel and control groups were prepared in the same way as for the scratch wound healing assay. The formation of a capillary-like structure was imaged after 12 h. The number of nodes and tube length of the capillary-like structure were evaluated by Image J software with an angiogenesis analysis module. 

### 4.5. Murine Wound Healing Models 

All mice were housed in the National Facility for Protein Science in Shanghai, with institution-approved animal care guidelines. All procedures were approved by the Institutional Animal Care and Use Committee of ShanghaiTech University (IACUC-20230621001). 

Deep second-degree burns and full-thickness excisional wounds were created on the dorsum of 10 to 12-week-old female ICR mice (GemPharmatech, Nanjing, China) [23,36]. The mice were anesthetized with 1.5% isoflurane (RWD, Shenzhen, China) and administered meloxicam (5 mg/kg, Solarbio, Beijing, China) subcutaneously for pain prophylaxis before surgery. The dorsal surface of the mice was shaved and two wounds were created on either side of the midline for each mouse. For the burn wound model, dorsal skin was scalded for 20 s with aluminum rods (6 mm diameter) which were pre-heated in boiling water. Then, the wounding area was immediately placed in cool water for 1 min to stop the continuous burn. Two days after burning, the wounds were debrided by using an 8 mm-diameter biopsy punch (Integra Miltex, Plainsboro, NJ, USA). For the excisional wound model, two full-thickness defects were created on the dorsal by using an 8 mm-diameter biopsy punch. All wounds were stented with donut-shaped silicone splints to prevent wound contraction. Three treating groups were randomly applied to the wounds: (a) untreated control, (b) HA hydrogel (0.5% *w*/*v*), and (c) HA-P hydrogel (0.5% *w*/*v*, with 0.2 mg/mL PR1P) (n = 6–10 wounds per group). For the hydrogel treatment group, 30 μL of gelling precursor was injected into the wound and exposed to 30 s of UV radiation to form a hydrogel dressing. Finally, all the wounds were covered by a commercially available transparent film dressing (Cofoe, Changsha, China). The digital images of the wounds were captured every other day, and the percentage of the original wound area was calculated by using Image J software.

### 4.6. Post-Surgery Analysis for In Vivo Studies

Animals were euthanized to harvest the wound tissues at day 14 post-wounding. Half of the specimens were fixed in 4% paraformaldehyde (Adamas Life, Shanghai, China), followed by paraffin embedding and processing. The blocked samples were sectioned into 7 μm slices perpendicular to the wound surface. The sections were stained with hematoxylin and eosin (H&E) and Masson’s trichrome (MT) (Solarbio, Beijing, China). Image J software was used to quantify the epidermis thickness and collagen density. The immunofluorescent staining of CD31 (1:200, ab182981, Abcam, Cambridge, UK) and α-SMA (1:200, ab5694, Abcam) followed the standard protocol. The fluorescence images were taken using a Zeiss LSM 710 confocal microscope (Carl Zeiss, Oberkochen, Germany) and stereological analysis was performed as previously described [41]. Moreover, another half of the tissue samples were snap-frozen for the Western blot assessment. Total protein was isolated from the tissue samples, separated on a 10% sodium dodecyl sulfate–polyacrylamide gel electrophoresis (SDS-PAGE) gel (Epizyme, Shanghai, China), and transferred to a polyvinylidene fluoride (PVDF) membrane (Merck Millipore, Darmstadt, Germany). Anti-Akt (1:3000, A18675, ABclonal, Wuhan, China), anti-Phospho-Akt (1:1000, 4060, Cell Signaling, Danvers, MA, USA), anti-VEGFA (1:1000, A12303, ABclonal, Wuhan, China), and anti-GAPDH (1:3000, AC002, ABclonal, Wuhan, China) were used as primary antibodies. An HRP-conjugated secondary antibody (1:3000, 7074P2, Cell Signaling, Danvers, MA, USA) was used to detect the signals with Immobilon ECL Ultra Western HRP Substrate (Millipore, Darmstadt, Germany). The intensity of the blots was calculated by using Image J software. 

### 4.7. Statistical Analysis

GraphPad Prism10 software was used for graphic illustrations and statistical analyses. All quantified data were shown as the mean ± standard deviation (SD) values. T-tests were used to compare means between the two groups. One-way ANOVA and two-way ANOVA were used to compare the significance among different groups. A value of *p* < 0.05 was considered statistically significant. 

## 5. Conclusions

In summary, we have described the development of an injectable hydrogel wound dressing based on photo-crosslinkable MeHA bonded with a pro-angiogenic peptide of PR1P. The in vitro assessments suggest the PR1P-bonded hydrogel increases VEGF recruitment and promotes cell migration and tubular formation. The in vivo studies demonstrate that the hydrogel wound dressing significantly enhances neovascularization and accelerates wound closure in both burn and excisional wound models, which can be related to the activation of the VEGF –Akt signaling pathway. Together, our data suggest this injectable hydrogel dressing indicates the potential for clinical applications in burns and other types of skin regeneration.

## Data Availability

All data associated with this study are available from the corresponding author upon reasonable request.

## Supplementary Materials

The following supporting information can be downloaded at: https://www.mdpi.com/article/10.3390/ijms25189948/s1.

## References

[1] Tottoli E.M., Dorati R., Genta I., Chiesa E., Pisani S., Conti B. (2020). Skin Wound Healing Process and New Emerging Technologies for Skin Wound Care and Regeneration. Pharmaceutics.

[2] Sun B.K., Siprashvili Z., Khavari P.A. (2014). Advances in skin grafting and treatment of cutaneous wounds. Science.

[3] Greenhalgh D.G. (2019). Management of Burns. N. Engl. J. Med..

[4] Radzikowska-Büchner E., Lopuszynska I., Flieger W., Tobiasz M., Maciejewski R., Flieger J. (2023). An Overview of Recent Developments in the Management of Burn Injuries. Int. J. Mol. Sci..

[5] Zwierello W., Piorun K., Skorka-Majewicz M., Maruszewska A., Antoniewski J., Gutowska I. (2023). Burns: Classification, Pathophysiology, and Treatment: A Review. Int. J. Mol. Sci..

[6] Goh M., Du M., Peng W.R., Saw P.E., Chen Z. (2024). Advancing burn wound treatment: Exploring hydrogel as a transdermal drug delivery system. Drug Deliv..

[7] Wang Y., Beekman J., Hew J., Jackson S., Issler-Fisher A.C., Parungao R., Lajevardi S.S., Li Z., Maitz P.K.M. (2018). Burn injury: Challenges and advances in burn wound healing, infection, pain and scarring. Adv. Drug Deliv. Rev..

[8] Barrientos S., Stojadinovic O., Golinko M.S., Brem H., Tomic-Canic M. (2008). Growth factors and cytokines in wound healing. Wound Repair Regen..

[9] Senturk B., Mercan S., Delibasi T., Guler M.O., Tekinay A.B. (2016). Angiogenic Peptide Nanofibers Improve Wound Healing in STZ-Induced Diabetic Rats. ACS Biomater. Sci. Eng..

[10] Chen Z., Wang L., Guo C., Qiu M., Cheng L., Chen K., Qi J., Deng L., He C., Li X. (2023). Vascularized polypeptide hydrogel modulates macrophage polarization for wound healing. Acta Biomater..

[11] Alves P.M., Fonseca D.R., Bidarra S.J., Gomes A., Gomes P., Barrias C.C., Martins M.C.L. (2024). Norbornene-chitosan nanoparticles with and without a conjugated VEGF-peptide analog to promote vascularization. Mater. Today Chem..

[12] Chen Y., Yuan Z.C., Sun W.Y., Shafiq M., Zhu J., Chen J.F., Tang H., Hu L., Lin W.K., Zeng Y.X. (2023). Vascular Endothelial Growth Factor-Recruiting Nanofiber Bandages Promote Multifunctional Skin Regeneration via Improved Angiogenesis and Immunomodulation. Adv. Fiber Mater..

[13] Sun Q., Silva E.A., Wang A., Fritton J.C., Mooney D.J., Schaffler M.B., Grossman P.M., Rajagopalan S. (2010). Sustained release of multiple growth factors from injectable polymeric system as a novel therapeutic approach towards angiogenesis. Pharm. Res..

[14] Scott R.C., Rosano J.M., Ivanov Z., Wang B., Chong P.L., Issekutz A.C., Crabbe D.L., Kiani M.F. (2009). Targeting VEGF-encapsulated immunoliposomes to MI heart improves vascularity and cardiac function. FASEB J..

[15] Golub J.S., Kim Y.T., Duvall C.L., Bellamkonda R.V., Gupta D., Lin A.S., Weiss D., Robert Taylor W., Guldberg R.E. (2010). Sustained VEGF delivery via PLGA nanoparticles promotes vascular growth. Am. J. Physiol. Heart Circ. Physiol..

[16] D’Andrea L.D., Iaccarino G., Fattorusso R., Sorriento D., Carannante C., Capasso D., Trimarco B., Pedone C. (2005). Targeting angiogenesis: Structural characterization and biological properties of a de novo engineered VEGF mimicking peptide. Proc. Natl. Acad. Sci. USA.

[17] Adini A., Adini I., Chi Z.L., Derda R., Birsner A.E., Matthews B.D., D’Amato R.J. (2017). A novel strategy to enhance angiogenesis in vivo using the small VEGF-binding peptide PR1P. Angiogenesis.

[18] Cao W., Zhang H., Zhou N., Zhou R., Zhang X., Yin J., Deng J., Ao X., Shi C. (2023). Functional recovery of myocardial infarction by specific EBP-PR1P peptides bridging injectable cardiac extracellular matrix and vascular endothelial growth factor. J. Biomed. Mater. Res. A.

[19] Yuan Z., Sheng D., Jiang L., Shafiq M., Khan A.U.R., Hashim R., Chen Y., Li B., Xie X., Chen J. (2022). Vascular Endothelial Growth Factor-Capturing Aligned Electrospun Polycaprolactone/Gelatin Nanofibers Promote Patellar Ligament Regeneration. Acta Biomater..

[20] Yuan Z.C., Shafiq M., Zheng H., Zhang L.X., Wang Z.W., Yu X., Song J.H., Sun B.B., El-Newehy M., El-Hamshary H. (2023). Multi-functional fibrous dressings for infectious injury treatment with anti-adhesion wound healing. Mater. Des..

[21] Burdick J.A., Chung C., Jia X., Randolph M.A., Langer R. (2005). Controlled degradation and mechanical behavior of photopolymerized hyaluronic acid networks. Biomacromolecules.

[22] Pailler-Mattei C., Bec S., Zahouani H. (2008). In vivo measurements of the elastic mechanical properties of human skin by indentation tests. Med. Eng. Phys..

[23] Dong Y.X., Cui M.H., Qu J., Wang X.C., Kwon S.H., Barrera J., Elvassore N., Gurtner G.C. (2020). Conformable hyaluronic acid hydrogel delivers adipose-derived stem cells and promotes regeneration of burn injury. Acta Biomater..

[24] Kakehi K., Kinoshita M., Yasueda S. (2003). Hyaluronic acid: Separation and biological implications. J. Chromatogr. B Analyt. Technol. Biomed. Life Sci..

[25] Stern R., Asari A.A., Sugahara K.N. (2006). Hyaluronan fragments: An information-rich system. Eur. J. Cell Biol..

[26] Sattar A., Rooney P., Kumar S., Pye D., West D.C., Scott I., Ledger P. (1994). Application of angiogenic oligosaccharides of hyaluronan increases blood vessel numbers in rat skin. J. Investig. Dermatol..

[27] Gao Y.R., Wang R.P., Zhang L., Fan Y., Luan J., Liu Z., Yuan C. (2023). Oral administration of hyaluronic acid to improve skin conditions via a randomized double-blind clinical test. Skin Res. Technol..

[28] Burdick J.A., Prestwich G.D. (2011). Hyaluronic acid hydrogels for biomedical applications. Adv. Mater..

[29] Litwiniuk M., Krejner A., Grzela T. (2016). Hyaluronic Acid in Inflammation and Tissue Regeneration. Wounds Compend. Clin. Res. Pract..

[30] Zhang Q.H., Yan K., Zheng X.Q., Liu Q.P., Han Y., Liu Z.G. (2024). Research progress of photo-crosslink hydrogels in ophthalmology: A comprehensive review focus on the applications. Mater. Today Bio.

[31] Ma H., Peng Y., Zhang S.N., Zhang Y.X., Min P.R. (2022). Effects and Progress of Photo-Crosslinking Hydrogels in Wound Healing Improvement. Gels.

[32] Johnson R.M., Richard R. (2003). Partial-thickness burns: Identification and management. Adv. Skin Wound Care.

[33] Ferrara N. (2004). Vascular endothelial growth factor: Basic science and clinical progress. Endocr. Rev..

[34] Adini A., Wu H., Dao D.T., Ko V.H., Yu L.J., Pan A., Puder M., Mitiku S.Z., Potla R., Chen H. (2020). PR1P Stabilizes VEGF and Upregulates Its Signaling to Reduce Elastase-induced Murine Emphysema. Am. J. Respir. Cell Mol. Biol..

[35] Tavares Pereira D.D.S., Lima-Ribeiro M.H.M., De Pontes-Filho N.T., Carneiro-Leão A.M.d.A., Correia M.T.d.S. (2012). Development of Animal Model for Studying Deep Second-Degree Thermal Burns. BioMed Res. Int..

[36] Dong Y.X., Sigen A., Rodrigues M., Li X.L., Kwon S.H., Kosaric N., Khong S., Gao Y.S., Wang W.X., Gurtner G.C. (2017). Injectable and Tunable Gelatin Hydrogels Enhance Stem Cell Retention and Improve Cutaneous Wound Healing. Adv. Funct. Mater..

[37] Volk S.W., Bohling M.W. (2013). Comparative wound healing--are the small animal veterinarian’s clinical patients an improved translational model for human wound healing research?. Wound Repair Regen..

[38] Shupp J.W., Nasabzadeh T.J., Rosenthal D.S., Jordan M.H., Fidler P., Jeng J.C. (2010). A Review of the Local Pathophysiologic Bases of Burn Wound Progression. J. Burn Care Res..

[39] He S.Q., Walimbe T., Chen H.Y., Gao K.W., Kumar P., Wei Y.F., Hao D.K., Liu R.W., Farmer D.L., Lam K.S. (2022). Bioactive extracellular matrix scaffolds engineered with proangiogenic proteoglycan mimetics and loaded with endothelial progenitor cells promote neovascularization and diabetic wound healing. Bioact. Mater..

[40] Luo J., Shi X., Lin Y., Yuan Y., Kural M.H., Wang J., Ellis M.W., Anderson C.W., Zhang S.M., Riaz M. (2021). Efficient Differentiation of Human Induced Pluripotent Stem Cells into Endothelial Cells under Xenogeneic-free Conditions for Vascular Tissue Engineering. Acta Biomater..

[41] Dong Y.X., Hassan W.U., Kennedy R., Greiser U., Pandit A., Garcia Y., Wang W.X. (2014). Performance of an in situ formed bioactive hydrogel dressing from a PEG-based hyperbranched multifunctional copolymer. Acta Biomater..

