# Supplementary material for: Photo-Crosslinked Pro-Angiogenic Hydrogel Dressing for Wound Healing
Source: Int J Mol Sci. 2024 Sep 15;25(18):9948. doi: 10.3390/ijms25189948 (PMC11432402; doi:10.3390/ijms25189948)
Supplement: Supplementary file 1 [file ijms-25-09948-s001.zip › ijms-3159675-supplementary.pdf]

Supporting Information

Photo-crosslinked Pro-angiogenic Hydrogel Dressing for Wound Healing

Wang Zhang,[1,2,3] Shuyi Qian,[1,2,3] Jia Chen,[1,2] Tianshen Jian,[1,2,3] Xuechun Wang,[1,3] Xianmin Zhu,[1,2,4] Yixiao Dong,[1,2,4*] Guoping Fan, [1,2*]

1. Shanghai Institute for Advanced Immunochemical Studies (SIAIS), ShanghaiTech University, Shanghai 201210, China; zhangwang@shanghaitech.edu.cn (W.Z.); qianshy@shanghaitech.edu.cn (S.Q.); chenjia1@shanghaitech.edu.cn (J.C.); jiantsh@shanghaitech.edu.cn (T.J.);
wangxch2@shanghaitech.edu.cn (X.W.); zhuxm@shanghaitech.edu.cn (X.Z.)
2. Shanghai Clinical Research and Trial Center, ShanghaiTech University, Shanghai 201210, China.
3. School of Life Science and Technology, ShanghaiTech University, Shanghai 201210, China.
4. Shanghai Academy of Sciences & Technology Institute of Model Animals Transformation, Shanghai 201203, China.

Correspondence: Email to: dongyx@shanghaitech.edu.cn (Y.D.);
fangp@shanghaitech.edu.cn (G.F.)

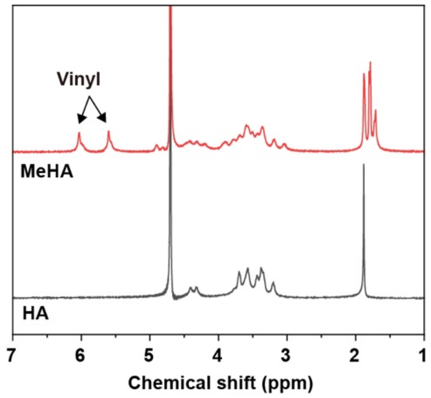

Figure S1. ^{1}H NMR spectra of methacrylated HA (MeHA) and HA polymers. The spectrum shows the vinyl group within the structure of MeHA at the chemical shifts between 6.4 and 5.8 ppm.

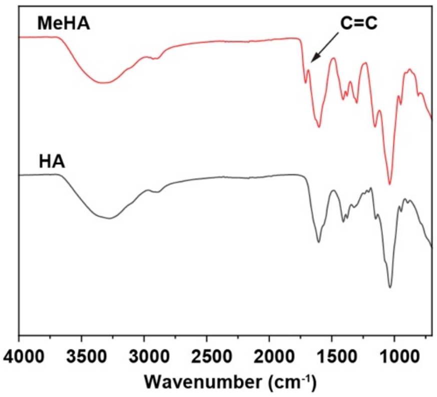

Figure S2. The FTIR spectra of methacrylated HA (MeHA) and HA polymers. The arrow labels the characterized "C=C" bond on the MeHA.

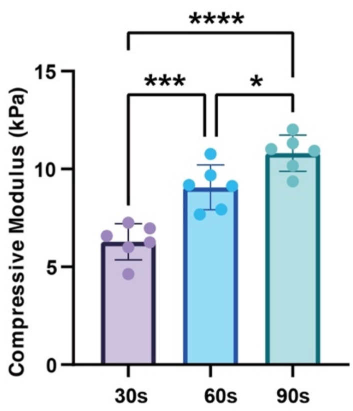

Figure S3. Compression test of HA hydrogels (0.5% *w/v*) with the 30 s, 60 s, and 90 s of UV exposure. (mean ± SD, n = 6, *$p < 0.05$, ***$p < 0.001$, ****$p < 0.0001$).

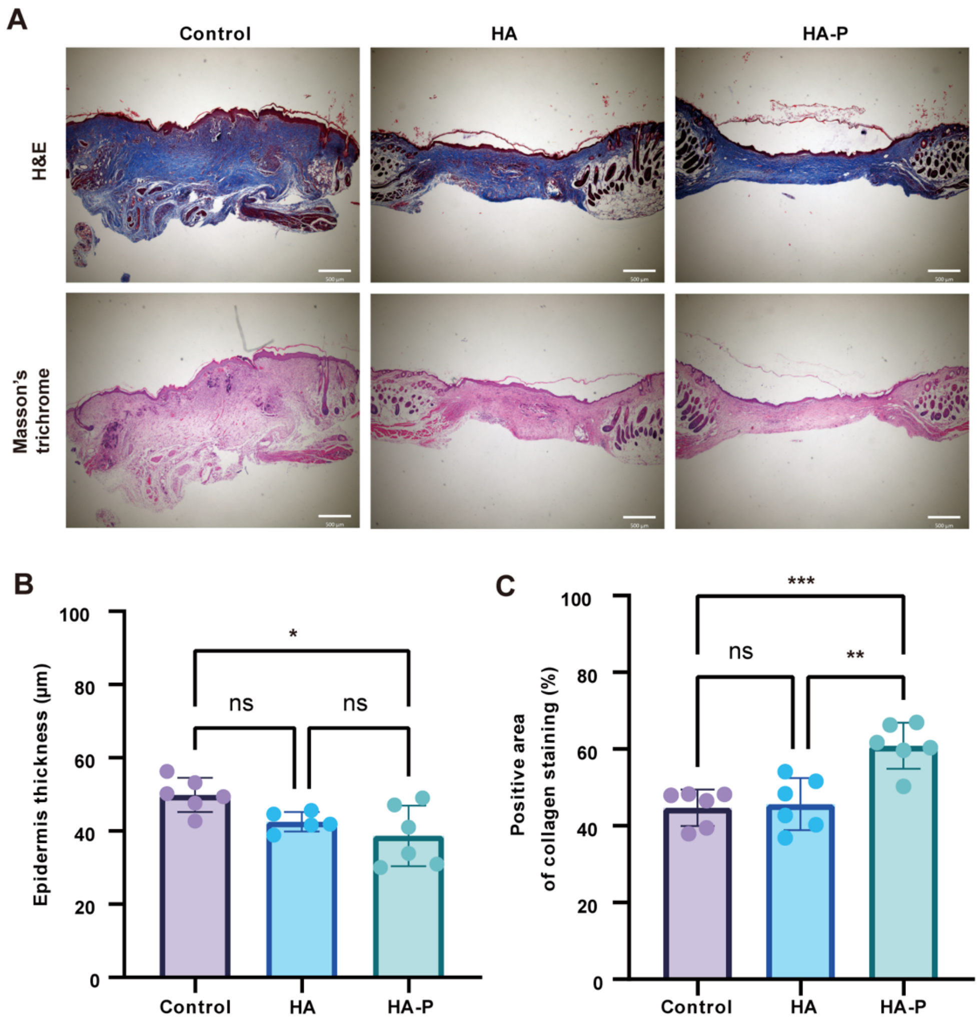

Figure S4. HA-P hydrogel promoted re-epithelialization and collagen deposition in full-thickness excisional wounds. (A) Representative images of H&E staining and Masson's trichrome staining of wounds at 14 days post wounding. (C) Quantitative analysis of epithelium thickness and (D) collagen density. (mean ± SD, n = 6, *$p < 0.05$, **$p < 0.01$, ***$p < 0.001$, ns, not statistically significant, scale bar, 500 μm).
